# Cross-sectional survey of malaria prevalence in tsunami-affected districts of Aceh Province, Indonesia

**DOI:** 10.1186/1865-1380-5-11

**Published:** 2012-02-21

**Authors:** David Muriuki, Sigrid Hahn, Braden Hexom, Richard Allan

**Affiliations:** 1 The MENTOR Initiative, La Prade11150 Villasavary France; 2 Mount Sinai School of Medicine, Department of Emergency Medicine, Box 1149, One Gustave L. Levy Place, New York, NY, 10029, USA

## Abstract

**Background:**

Malaria is endemic to Indonesia. However, there are few prevalence data available from Aceh Province because of the long-standing separatist conflict and decentralization of the public health system. The Mentor Initiative, which specializes in malaria control in humanitarian emergencies, was one of the non-governmental organizations to respond to the 2004 Indian Ocean tsunami in Aceh. Data on malaria prevalence were gathered to guide and evaluate programmatic efforts.

**Findings:**

The Mentor Initiative conducted community-based malaria prevalence surveys in 2005 and 2006 in five districts along the tsunami-affected western coastline. A total of 11,763 individuals in 3,771 households were tested. The overall slide positivity rate in 2005 and 2006 for all *Plasmodium *species was 2.1% (*n *= 252, 95% CI 1.9%-2.4%). Slide positivity rates ranged from 0 to 55% among villages. Overall, 57% of the 252 cases were infected with *P. falciparum *(*n *= 144, 95% CI 51.0%-63.3%), and 40.1% were infected with *P. vivax *(*n *= 101, 95% CI 34.0%-46.1%), with 0.03% (*n *= 7, 95% CI 0.8%-4.8%) being mixed infections. Males were significantly more likely to be affected than females (2.8% vs 1.5%, *p *< 0.01). Infection was more common in those over the age of 5 (2.3% vs. 0.6%, *p *< 0.01).

**Conclusions:**

Local prevalence data are needed to design effective community-based malaria control programs, as endemicity varies greatly within districts. Certain villages were found to be hyperendemic, with slide positivity rates far higher than average in Indonesia. There is a need for ongoing malaria surveillance in Aceh Province to monitor prevention and treatment efforts.

## Introduction

Malaria is endemic throughout much of Indonesia. The Indonesian government reported a countrywide malaria prevalence of 850 per 100,000 in 2001 [1]. The World Health Organization's (WHO) 2008 World Malaria Report stated that 37% of Indonesia's population lived in a high transmission area (≥ 1 case/1,000), 14% lived in a low transmission area (0-1 cases/1,000), and 50% lived in a malaria-free area [2]. Endemicity tends to be higher on the more heavily forested outer islands. Approximately 46% of malaria infections in Indonesia are due to *Plasmodium falciparum *[3].

Sumatra, one of Indonesia's outer islands, was severely affected by the 2004 Indian Ocean tsunami, with much of the destruction located in the northern Aceh Province. Aceh Province had been quite isolated prior to the tsunami because of a long-standing separatist conflict, and therefore relatively little malaria prevalence information is publicly available. The South-East Asia Regional Office of the WHO reported that Aceh had low to moderate endemicity in 2005 [4]. The Indonesian Ministry of Health reported fewer than 1,000 blood smears and rapid diagnostic tests (RDTs) in Aceh were positive for malaria in 2002, but was unable to provide a slide positivity rate [5]. Between 5,000 and 9,999 "clinically confirmed" cases were reported in Aceh during the same year, but "clinical confirmation" is known to be inaccurate [5]. The World Malaria Report found that much of Sumatra had a prevalence ranging from 1 to 100 reported cases per 1,000, but reported no data from Aceh Province [1].

The Mentor Initiative, a non-governmental organization specializing in malaria prevention and treatment in complex emergencies, was one of many humanitarian groups that provided assistance in Indonesia after the tsunami. Given the paucity of baseline data on malaria prevalence, and the need to set prevention priorities and monitor program effectiveness, the Mentor Initiative conducted two community-based malaria prevalence surveys in tsunami-affected subdistricts of Aceh Province in 2005 and 2006. The data presented here are the first published in a peer-reviewed journal from Aceh, and this is among the few publications of the state of malaria prevalence in the post-tsunami setting.

## Methods

### Survey protocol

Although rain can occur episodically throughout the year, the rainy season in Aceh usually lasts from September to February. Malaria transmission is thought to vary seasonally in Aceh, peaking during the rainy season. Surveys were conducted during the dry season between May and July 2005 and again between April and July 2006.

Aceh province is divided into 21 districts. Five tsunami-affected districts were identified for the community-based malaria prevalence surveys conducted as part of a broader malariometric survey: Aceh Barat, Aceh Jaya, Nagan Raya, Woyla Barat, and Simeulue. Selection was based, in part, on predicted malaria burden, and also on the area's accessibility and security. In 2005, seven subdistricts of the aforementioned districts were surveyed: Seulimeum, Lamno, Woyla Induk, Woyla Timur, Beutong, Simeulue Timur, and Teupah Selatan. In 2006, a follow-up survey was conducted in these same subdistricts (except that Sungai Mas in Woyla Barat district was included and Simeulue Timur was excluded). Both years' data are presented in this article.

Within each subdistrict, Probability Proportional to Size cluster sampling was used to select villages. Within each village, households were selected by simple random sampling. The target sample size was 10% of the population of each subdistrict. Written consent was obtained from the head of each household, and all members of the household were surveyed.

In each subdistrict, survey data were collected by two teams of three health care providers (usually nurses) from the local clinics (*puskesmus*), and data collection was overseen by a supervising physician from The Mentor Initiative. The survey team was trained to collect survey data, perform the RDT, and prepare the thick and thin blood smears for microscopy. All previously untreated patients who tested positive for malaria were given appropriate therapy according to local and Roll Back Malaria in Complex Emergency guidelines.

### Laboratory diagnosis

Microscopy was considered the gold standard for diagnosis of malaria infection. Slides were prepared by survey staff and read by a Mentor-trained laboratory technician as well as two technicians from the Provincial Health Office. Systematic external quality controls were conducted to ensure accuracy of the blood smear readings by the survey team. The prevalence survey was conducted contemporaneously with a second study designed to field test RDTs. In 2006, the RDT used was Falcivax, which detects *P. falciparum, P. vivax*, and mixed infections. During 2006, the blood smears obtained in the subdistricts Woyla Induk and Woyla Timur were contaminated during preparation and rendered unreadable. Therefore, in these two subdistricts, RDT results are reported in lieu of blood smear results.

### Statistical analysis

Data were entered into Epi Data and analyzed using Epi Info. Pearson's chi-square test was used to test for significance, where appropriate. *P *values of < 0.05 were considered statistically significant.

### Ethical considerations

Approval to carry out the survey was obtained by The Mentor Initiative from the Provincial Health Office and each District Health Office in Aceh Province. The Institutional Review Board of Mount Sinai School of Medicine approved the retrospective data analysis.

## Results

A total of 3,771 households and 11,763 individuals were surveyed in 220 villages in 2005 and 2006. Details of individuals, households, and villages surveyed are provided in Table 1. Overall, 43.6% (*n *= 5,130) of those surveyed were male. Males were significantly more likely to have parasitemia than females (2.8% vs. 1.5%, *p *< 0.01). Of the population surveyed, 12.3% (*n *= 1,447) was less than or equal to 5 years old and 23.9% (*n *= 2,817) less than or equal to 10. The mean age could not be calculated as data were recorded categorically (by age group). Parasitemia was less common in children less than or equal to 5 years old compared with older children and adults (0.6% vs. 2.3%, *p *< 0.01). Malaria infection was significantly more common among actively febrile patients (16% vs. 1.7%, *p *< 0.01).

**Table 1:** Number of villages, households, and individuals surveyed in each subdistrict

	Villages		Households		Individuals	
Subdistrict	2005	2006	2005	2006	2005	2006
Seulimeum	19	24	285	480	903	1791
Lamno	24	24	360	408	1070	944
Woyla Induk	20	22	397	308	1184	1012
Woyla Timor	13	13	240	104	775	257
Sungai Mas	-	9	-	81	-	219
Beutong	10	14	250	350	1189	1026
Simeulue Timur	10	-	150	-	458	-
Teupah Selatan	10	8	150	208	392	543
**Total**	**106**	**114**	**1832**	**1939**	**5971**	**5792**

**Figure 1:**
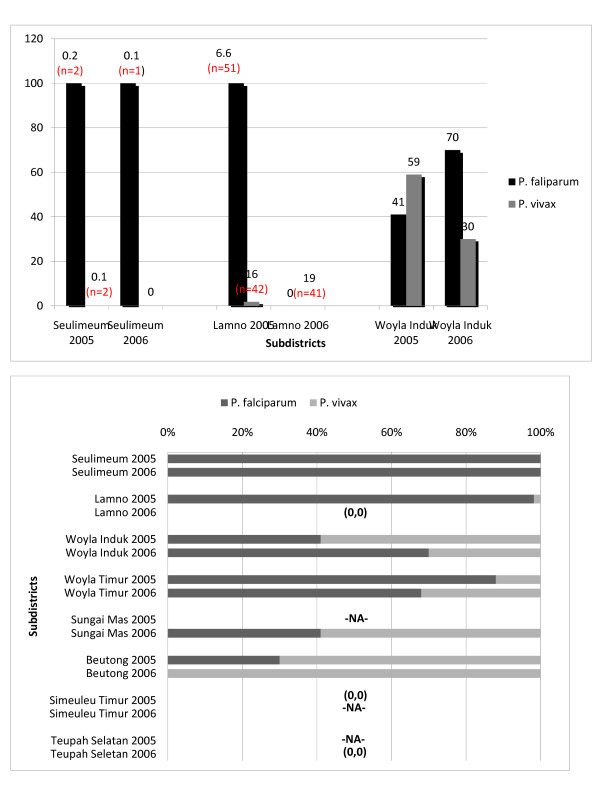
**Positive blood smears**. **(a) **Percent of positive blood smears in 2005 and 2006, by subdistrict. **(b) **Percent of blood smears positive for *P. falciparum *and *P. vivax *in each subdistrict.

Prevalence data and slide positivity rates for *Plasmodium falciparum *versus *P. vivax *are presented in Figure 1a, b. The overall slide positivity rate in both 2005 and 2006 for all *Plasmodium *species was 2.1% (*n *= 252, 95% CI 1.9%-2.4%), but it was much higher in the subdistricts of Woyla Timur and Sungai Mas (Figure 1a). Two species of malaria parasites, *Plasmodium falciparum *and *P. vivax*, were identified. Overall, *P falciparum *accounted for 57.1% of infections (*n *= 144, 95% CI 51.0%-63.3%) and *P. vivax *for 40.1% (*n *= 101, 95% CI 34.0%-46.1%), with 0.03% (*n *= 7, 95% CI 0.8%-4.8%) being mixed infections. The proportion of *P. falciparum *and *P. vivax *varied among subdistricts. As displayed in Figure 1b, *P. falciparum *was more common in the subdistricts of Woyla Induk and Woyla Timur, whereas *P. vivax *predominated in Beutong and Sungai Mas. As discussed in the methods section, the RDT Falcivax, which can distinguish among *P. falciparum, P. vivax*, and mixed infections, was substituted for blood smear results in 2006 in the subdistricts of Woyla Induk and Woyla Timur because of contamination of the slides.

Within each subdistrict, the intervillage positivity rates varied widely (see Table 2). In the subdistricts of Woyla Induk and Woyla Timur, some villages in 2005 had rates as high as 13% and 31%, respectively. In 2006, villages in the subdistricts of Woyla Induk, Woyla Timur, and Sungai Mas had positivity rates of 26%, 41%, and 55%, respectively.

**Table 2:** Prevalence range among villages within each subdistrict

	Prevalence range among villages (%)	
Subdistrict	2005	2006
Seulimeum	0-2.2	0-1.3
Lamno	1-1.5	0
Woyla Induk	0-13	0-26
Woyla Timur	0-31	0-41
Sungai Mas	n/a	0-55
Beutong	0-7	0-4
Simeulue Timur	0	n/a
Teupah Selatan	0	0

## Discussion

This is the first peer-reviewed study of malaria prevalence in post-tsunami Aceh Province, Indonesia. Most of what is known about malaria prevalence in Indonesia is based in regions of the country outside of Aceh and therefore has a limited application in Aceh itself [2]. Our results show great variability of malaria prevalence among subdistricts, and even more so among villages, with small areas of transmission rates approaching those found in sub-Saharan Africa [1].

Because of the limited data available prior to the 2004 Indian Ocean tsunami, it is difficult to compare malaria prevalence before and after the disaster. The post-tsunami conditions were thought to be high risk for a malaria epidemic, with massive displacement of a semi- or non-immune population, temporary and crowded housing, standing water with the creation of new vector breeding areas, and disruption of the public health and health care system. These data strongly suggest that an epidemic did not occur and that malaria transmission was kept at low rates in most areas during the 2 years following the disaster. However, without pre-tsunami data, the impact of the massive malaria control efforts by various national and international agencies cannot be determined from this study.

Our survey found that Aceh males are more likely to have parasitemia than females, which is consistent with the Indonesian Ministry of Health's report that death rates for men are 11 per 100,000 compared with 8 per 100,000 for females [3]. This may reflect variable exposure to mosquitoes through differing occupational or recreational activities. We also found great variation in malaria prevalence rates between and within subdistricts. Several studies have examined the spatiotemporal variations in malaria epidemic risk, particularly in Africa [6,7]. The wide variability in endemicity among villages likely reflects the microclimate of that village, with variable forest cover and water sources. Factors that serve as important determinants of the severity of malaria transmission include altitude, forest cover, soil water holding capacity, and precipitation. Despite their close proximity, differences in these risk factors among the subdistricts might impact the intensity of malaria transmission. Incorporating such information into future malaria prevention and control programs could serve to predict the likelihood of severe malaria epidemics and allow for local or community-based control efforts to be tailored appropriately.

## List of abbreviations

CI: confidence interval; WHO: World Health Organization; RDT: rapid diagnostic tests.

## Competing interests

The authors declare that they have no competing interests.

## Authors' contributions

DM helped conceive, design, and coordinate the study in the field. SH performed the statistical analysis and co-wrote the manuscript. BH performed the statistical analysis and co-wrote the manuscript. RA conceived of the study, participated in its design and coordination and reviewed the manuscript. All authors read and approved the final manuscript.
